# Evaluating non-linear thresholds and synergistic interactions in the comorbidity of preoperative insomnia and emergence agitation: a SHAP-based machine learning study

**DOI:** 10.3389/fneur.2026.1875540

**Published:** 2026-09-14

**Authors:** Jianheng Zhao, Xiaoxu Yu, Bangjian Zhang, Mengying Wang, Xiaoyan Yang

**Affiliations:** Panzhihua Central Hospital, Panzhihua, China

**Keywords:** comorbidity, emergence agitation, machine learning, preoperative insomnia, SHAP

## Abstract

**Background:**

Preoperative insomnia disorder (PID) and emergence agitation (EA) often present as a complex perioperative comorbidity. Traditional linear models may not adequately capture their underlying interactive mechanisms.

**Methods:**

This retrospective, single-center study analyzed 542 adult surgical patients. Machine learning algorithms, particularly XGBoost, were developed using stratified random splitting (70% training, 30% testing) to predict the PID-EA co-occurrence. The Shapley Additive exPlanations (SHAP) framework was utilized to interpret statistical associations and non-linear interactions among clinical variables.

**Results:**

The XGBoost model demonstrated superior discriminative performance (AUC = 0.874 [95% CI: 0.812–0.936], AUPRC = 0.883, Brier score = 0.150) compared to logistic regression (AUC = 0.749 [95% CI: 0.675–0.823]). SHAP analysis indicated that preoperative cortisol and melatonin were the primary features associated with the model’s predictions. Partial dependence plots suggested potential non-linear risk thresholds for neuroendocrine markers. Furthermore, interaction analyses illustrated that severe postoperative pain may synergistically amplify the risk associated with preoperative insomnia.

**Conclusion:**

The integration of XGBoost and SHAP offers an interpretable approach to evaluating the PID-EA comorbidity network. These findings highlight potential clinical thresholds and interaction patterns, warranting further validation through multi-center prospective studies.

## 1 Introduction

Emergence agitation (EA) is a challenging, often distressing complication of general anesthesia in adults and can be linked to a number of adverse postanesthetic outcomes such as self-extubation, bleeding, and increased length of stay (1). In parallel, surgical patients experience high rates of insomnia, which is increasingly acknowledged as a risk factor for perioperative neurocognitive and behavioral disorders, a condition defined as preoperative insomnia disorder (PID) (2). Observations from clinical practice suggest that there is often a correlation between the clinical events of PID and EA, indicating a possible “comorbidity” phenotype (3).

The association of EA with PID indicates that there may be common pathophysiological mechanisms (4, 5). This comorbid association may be mediated by factors such as hypothalamic–pituitary–adrenal (HPA) axis dysfunction, systemic inflammation, and disturbances in the circadian rhythm, which are associated with clinical biomarkers including serum cortisol, neutrophil-to-lymphocyte ratio (NLR), and melatonin (6). In addition, underlying factors of PID may increase the risk of EA, while perioperative factors such as surgical time, intraoperative hemodynamics, and postoperative pain may also contribute to this risk (7, 8). The specific ways in which these multi-dimensional factors interact, which are proposed as possible shared vulnerability pathways rather than established mechanisms, however, remain not fully known (9).

Existing clinical prediction models tend to rely on traditional linear statistical techniques (e.g., logistic regression) (10, 11). These traditional methods are based on the assumption that the relationships between variables are linear and independent of each other, and they may not be able to capture the complex and synergistic relationships that are typical of clinical comorbidity networks (12). High-performing machine learning algorithms can efficiently handle medical data with high dimensionality and non-linear structures, but their use in clinical settings is often limited by the opacity of advanced algorithms (13). To overcome this deficiency, a methodological approach is needed; Shapley Additive exPlanations (SHAP) provides local and global interpretability for complex algorithmic models by visualizing potential nonlinear predictive associations and interaction patterns (14).

Thus, this study intends to explore the interaction mechanisms of the clinical comorbidity between PID and EA among adult patients undergoing general anesthesia (GA). This study uses multiple machine learning techniques and utilizes the SHAP framework to help interpret the possible non-linear relationships, predictive cutoffs, and model-based interaction patterns of pre- and peri-operative variables. The goal is to offer an analysis tool that can be interpreted and potentially guide more specific clinical care for this clinical phenotype.

## 2 Materials and methods

### 2.1 Study design and ethical approval

A retrospective observational study was performed by collecting clinical data from the electronic medical record system from January 2021 to December 2023. The study protocol was approved by the Institutional Review Board (approval number: ZXYYREC-2026-092) of the Panzhihua Central Hospital. All experiments with human subjects were conducted with the approval of the institutional and national research committees and in accordance with the Declaration of Helsinki as revised in 1975. In this retrospective study, the need for written informed consent was waived by the Institutional Review Board because of the use of de-identified patient information.

### 2.2 Patient selection

The inclusion criteria were defined as follows: (1) adult patients aged 18 years or older; (2) scheduled for elective surgery under general anesthesia; and (3) completion of standardized preoperative sleep and psychological assessments, which are mandatory routine protocols for pre-anesthesia evaluation at our institution to minimize selection bias. Patients were excluded if they met any of the following criteria: (1) a documented history of severe psychiatric or neurological disorders; (2) regular preoperative use of sedative-hypnotic medications or antidepressants; (3) undergoing reoperation within 24 h; or (4) missing data for key clinical variables or laboratory measurements.

### 2.3 Data collection and clinical variables

The primary outcome was defined as the concurrent manifestation of preoperative insomnia disorder (PID) and postoperative emergence agitation (EA). PID was assessed using the Insomnia Severity Index (ISI, score ≥ 8), and EA was evaluated clinically during the post-anesthesia care unit recovery phase using the Riker Sedation-Agitation Scale (RSAS score ≥ 5).

A total of 16 multi-dimensional clinical features were systematically extracted as associative features, with the intended temporal evaluation point set at PACU discharge, acknowledging that postoperative variables (e.g., VAS, extubation time) occur concurrently with EA. Demographic data included age, body mass index (BMI), gender, and American Society of Anesthesiologists (ASA) physical status. Psychological and biological markers comprised the preoperative ISI score, preoperative Hospital Anxiety and Depression Scale for anxiety (HADS-A), preoperative serum cortisol (nmol/L), preoperative neutrophil-to-lymphocyte ratio (NLR), and preoperative melatonin (pg/mL). To ensure consistency regarding circadian fluctuations, all cortisol and melatonin measurements were derived from single fasting venous blood samples collected between 06:00 and 07:00 a.m. on the morning of the surgery, analyzed utilizing standard enzyme-linked immunosorbent assay (ELISA) platforms. Surgical and anesthetic parameters incorporated the duration of surgery (minutes), intraoperative sufentanil dose (μg/kg), intraoperative propofol dose (mg/kg), and the duration of intraoperative hypotension (minutes). Postoperative variables included extubation time (minutes), postoperative Visual Analog Scale (VAS) for pain, and the incidence of catheter-related bladder discomfort (CRBD).

### 2.4 Machine learning algorithms and model development

The dataset was split into two subsets: 70% for training and 30% for testing. To reduce dimensionality and possible multi-collinearity, the Random Forest Recursive Feature Elimination (RF-RFE) and the Least Absolute Shrinkage and Selection Operator (LASSO) algorithms were used to perform feature selection prior to modeling. Six different algorithms were tested: eXtreme Gradient Boosting (XGBoost), Random Forest (RF), Gradient Boosting Machine (GBM), Support Vector Machine (SVM), K-Nearest Neighbors (KNN), and Logistic Regression (LR) as the traditional baseline. This was performed using a 5-fold cross-validation method on the training set, where the optimal model (XGBoost) was selected strictly based on internal training performance, ensuring the test set remained completely independent for final evaluation only. For XGBoost, the objective function used was binary logistic regression, and parameters were optimized using grid search (e.g., maximum depth = 4, learning rate/eta = 0.1, number of rounds = 100). For the SVM algorithm, the radial basis function kernel was employed, and for the RF model, 500 decision trees were utilized.

### 2.5 Handling of class imbalance

During the training phase, algorithmic class weight adjustment techniques were used to address potential class imbalance between the comorbidity and non-comorbidity groups. More specifically, the positive class weight parameter (e.g., scale_pos_weight in XGBoost) was adjusted to match the ratio of negative to positive samples in the training set. This was only done with the training data to avoid data leakage. Furthermore, Precision-Recall (PR) curves were used as the main evaluation metric since they are a more informative way of evaluating the performance of the algorithms on minority positive classes.

### 2.6 Statistical analysis

The data were analyzed statistically using R software (Version 4.3.1). Normality of continuous variables was evaluated by using the Shapiro–Wilk test. One-way Analysis of Variance (ANOVA) was used to compare normally distributed continuous variables, which are expressed as mean ± standard deviation. Variables that are not normally distributed are presented as the median [interquartile range (IQR)] and analyzed using the Kruskal-Wallis test. Categorical variables are presented as frequencies (percentages) and were tested by the Chi-square test.

Model discrimination was assessed using the Area Under the Receiver Operating Characteristic Curve (AUC), and the pairwise DeLong test was used for statistical comparisons of ROC curves. Calibration plots and Brier scores were used to evaluate model calibration, with the addition of Loess smoothing to highlight departures from the reference line. Decision Curve Analysis (DCA) was used to assess clinical utility. SHapley Additive exPlanations (SHAP) framework was used to determine the marginal contribution, non-linear dependence and interaction among variables. A two-sided *p*-value < 0.05 was considered to indicate statistical significance.

## 3 Results

### 3.1 Baseline characteristics and feature selection

In all, 542 adult patients undergoing elective surgery under general anesthesia were analyzed. Using the pre-operative Insomnia Severity Index (ISI) and the presence of emergence agitation (EA), the group was divided into four clinical phenotypes: Control (*n* = 157), PID Only (*n* = 139), EA Only (*n* = 34) and Comorbid (*n* = 212). There were significant differences between the groups in several areas (summarized in Table 1). Based on Bonferroni-adjusted post hoc comparisons, stress markers (preoperative cortisol and neutrophil-to-lymphocyte ratio), intraoperative hypotension time and postoperative pain scores were also higher in the Comorbid group than in the other groups (adjusted *p* < 0.001) (note: The differences in preoperative ISI scores between groups are an expected artifact since ISI was utilized to define the PID status). However, the level of melatonin in the Comorbid group was lower before surgery.

**Table 1:** Baseline characteristics stratified by preoperative insomnia and emergence agitation comorbidity phenotypes.

Variables	Total (*n* = 542)	Control (PID−/EA−) (*n* = 157)	PID Only (PID+/EA-) (*n* = 139)	EA Only (PID−/EA+) (*n* = 34)	Comorbid (PID+/EA+) (*n* = 212)	*p*-value
Demographics						
Age (years), Mean ± SD	52.8 ± 12.5	51.9 ± 11.6	49.0 ± 12.1	60.0 ± 13.8	54.8 ± 12.3	< 0.001
BMI (kg/m^2^), Mean ± SD	24.4 ± 3.5	24.4 ± 3.5	24.4 ± 3.8	24.9 ± 2.7	24.3 ± 3.4	0.871
Gender (male), *n* (%)	278 (51.3%)	79 (50.3%)	72 (51.8%)	21 (61.8%)	106 (50.0%)	0.635
ASA status (II), *n* (%)	282 (52.0%)	81 (51.6%)	76 (54.7%)	15 (44.1%)	110 (51.9%)	0.604
ASA status (III), *n* (%)	144 (26.6%)	43 (27.4%)	39 (28.1%)	12 (35.3%)	50 (23.6%)	0.614
Psychological & biomarkers						
Preop ISI score, median [IQR]	10 [6–13]	5 [3–6]	11 [9–12]	6 [5–7]	14 [11–17]	< 0.001
Preop anxiety (HADS), median [IQR]	7 [4–9]	4 [3–6]	7 [6–9]	4 [2–5]	9 [7–11]	< 0.001
Preop cortisol (nmol/L), Mean ± SD	379.6 ± 58.0	331.9 ± 45.8	371.9 ± 41.9	376.9 ± 48.5	420.5 ± 46.3	< 0.001
Preop NLR (ratio), median [IQR]	3.03 [2.35–3.78]	2.42 [1.99–3.02]	2.56 [2.02–3.27]	3.58 [3.11–4.25]	3.64 [3.07–4.18]	< 0.001
Preop melatonin (pg/mL), Mean ± SD	13.9 ± 4.5	17.8 ± 3.2	13.8 ± 3.7	14.4 ± 3.4	11.0 ± 3.5	< 0.001
Surgical & anesthetic						
Surgery duration (mins), Mean ± SD	110 ± 43	111 ± 47	111 ± 41	105 ± 44	110 ± 42	0.913
Intraop sufentanil (μg/kg), Mean ± SD	0.40 ± 0.11	0.40 ± 0.11	0.40 ± 0.11	0.37 ± 0.08	0.40 ± 0.11	0.490
Intraop propofol (mg/kg), Mean ± SD	12.4 ± 4.6	12.6 ± 5.0	12.5 ± 4.5	11.8 ± 4.6	12.4 ± 4.5	0.800
Hypotension duration (mins), Median [IQR]	6 [0–12]	1 [0–6]	1 [0–5]	12 [7–22]	11 [8–15]	< 0.001
Postoperative						
Extubation time (mins), median [IQR]	17 [14–20]	17 [14–21]	16 [13–20]	17 [15–24]	17 [14–20]	0.547
Postop VAS score, median [IQR]	5 [3–7]	3 [2–5]	3 [2–4]	6 [5–9]	7 [6–8]	< 0.001
CRBD incidence (yes), *n* (%)	126 (23.2%)	29 (18.5%)	36 (25.9%)	4 (11.8%)	57 (26.9%)	0.084

The distribution overlap in the Venn diagram (Figure 1A) indicates that a substantial number of patients (212 patients had both EA and PID) may exhibit a comorbid association. Spearman correlation analysis (Figure 1B) showed that there is a complex network of interrelated clinical and biological features, highlighting the need for advanced dimensionality reduction.

**Figure 1:**
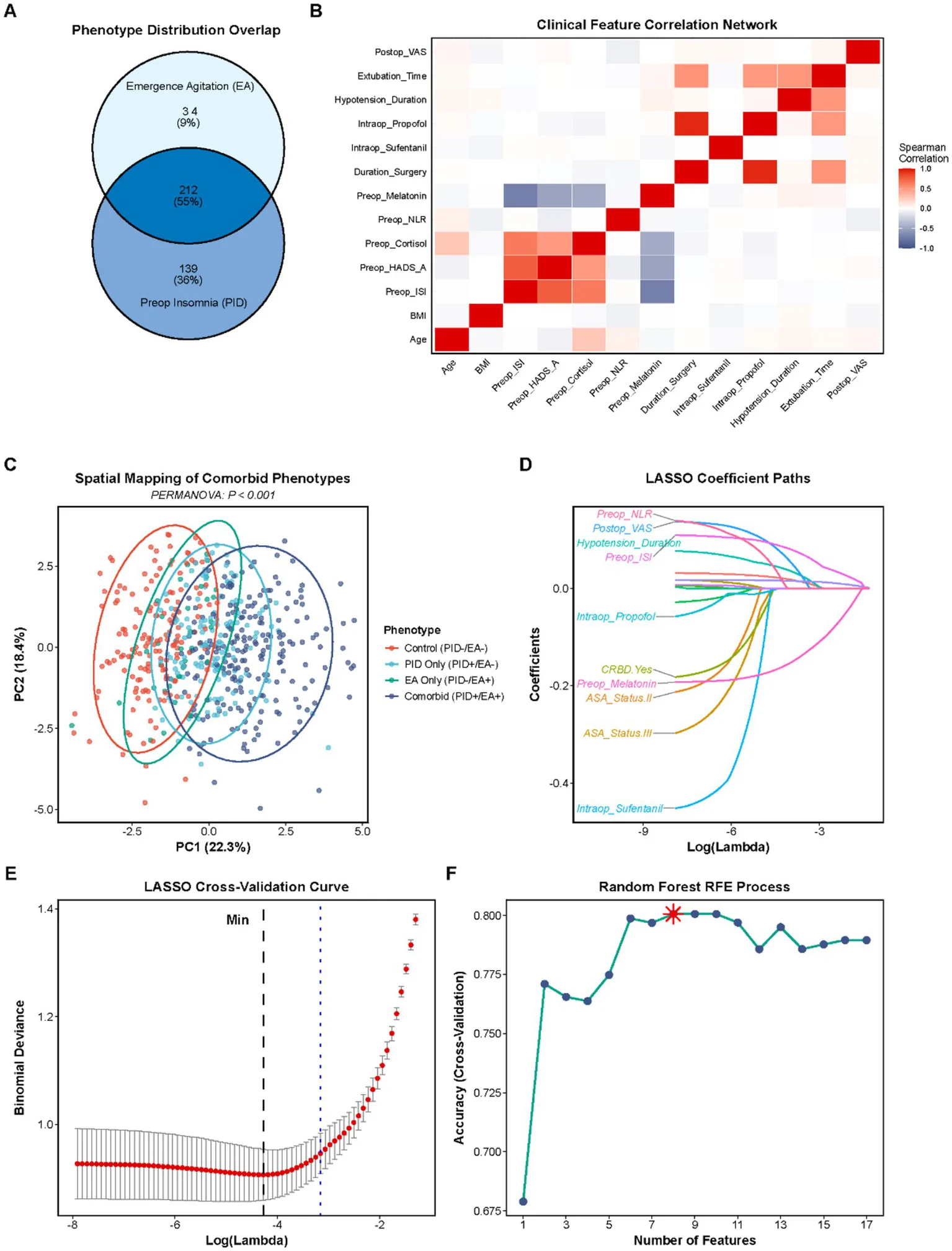
Feature selection and spatial mapping of comorbidity phenotypes. **(A)** Venn diagram showing the overlap distribution of preoperative insomnia and emergence agitation in the study cohort. **(B)** Heatmap illustrating the Spearman correlation network among clinical and biological features. **(C)** Principal component analysis (PCA) plot presenting the spatial distribution of the four clinical phenotypes. **(D)** LASSO coefficient paths of the clinical features. **(E)** LASSO cross-validation curve indicating the optimal penalty parameter (lambda). **(F)** Random forest recursive feature elimination (RF-RFE) process suggesting the optimal subset of features.

Principal Component Analysis (PCA) was used to characterize the spatial distribution of these phenotypes (Figure 1C). Results of the PERMANOVA test indicated statistical significance (*p* < 0.001); however, there was much overlap in 95 percent confidence ellipses among the four groups. This high spatial overlap suggests that a traditional linear dimensional reduction technique may not be able to distinguish the comorbid condition and further underscores the need to apply non-linear machine learning algorithms to model the complex underlying patterns of this comorbidity.

A systematic feature selection process was conducted to prevent multi-collinearity and avoid overfitting the model in subsequent steps. The LASSO regression algorithm was used to trace the coefficient paths (Figure 1D), and the optimal penalty parameter (lambda) was identified through cross-validation, which reduced redundant feature coefficients to zero (Figure 1E). Next, the Random Forest Recursive Feature Elimination (RF-RFE) method was used to assess the cross validation accuracy for different number of features. The accuracy of the evaluation curve was highest when 8 core features were kept, as shown in Figure 1F (red asterisk), a cutoff determined by identifying the accuracy plateau in conjunction with the “one standard error” rule during cross-validation. Although these exploratory algorithms indicated that mathematical redundancy could be reduced to 8 features, we purposefully retained all 16 original clinical features for the final downstream XGBoost modeling. This decision was made to preserve the comprehensive clinical architecture required for holistic SHAP interaction and physiological mapping.

### 3.2 Performance evaluation of machine learning models

Based on the need for non-linear algorithms that was identified during the spatial mapping phase, six different machine learning models were created to predict the risk of PID-EA comorbidity. They were systematically tested on the independent testing set for their prediction performance. The XGBoost model had the highest discriminative ability with an Area Under the Receiver Operating Characteristic Curve (AUC) of 0.874 [95% CI: 0.812–0.936] as presented in Figure 2A. The traditional Logistic Regression (LR) model had the lowest AUC (0.749 [95% CI: 0.675–0.823]) in comparison. The discriminatory ability of the two models was statistically different (*p* = 0.005) when evaluated by the DeLong test, suggesting a statistical performance advantage, which may be related to its capacity for modeling complex associations, although causality cannot be inferred. Furthermore, Precision-Recall (PR) curves were generated to assess the precision of the models, given the possible class imbalance in the dataset. The XGBoost model consistently outperformed the natural baseline prevalence (prevalence = 0.39, reflecting the true stratified proportion) in terms of Area Under the Precision-Recall Curve (AUPRC = 0.883) over the other models as shown in Figure 2B. To further conceptualize these differences, a spider web radar chart (see Figure 2C) was used to compare multi-dimensional performance metrics. The XGBoost model seemed to perform better than the LR model in all five dimensions evaluated, such as AUC, Accuracy, Sensitivity, Specificity, and F1-Score, which suggests that the XGBoost model can achieve more balanced classification.

**Figure 2:**
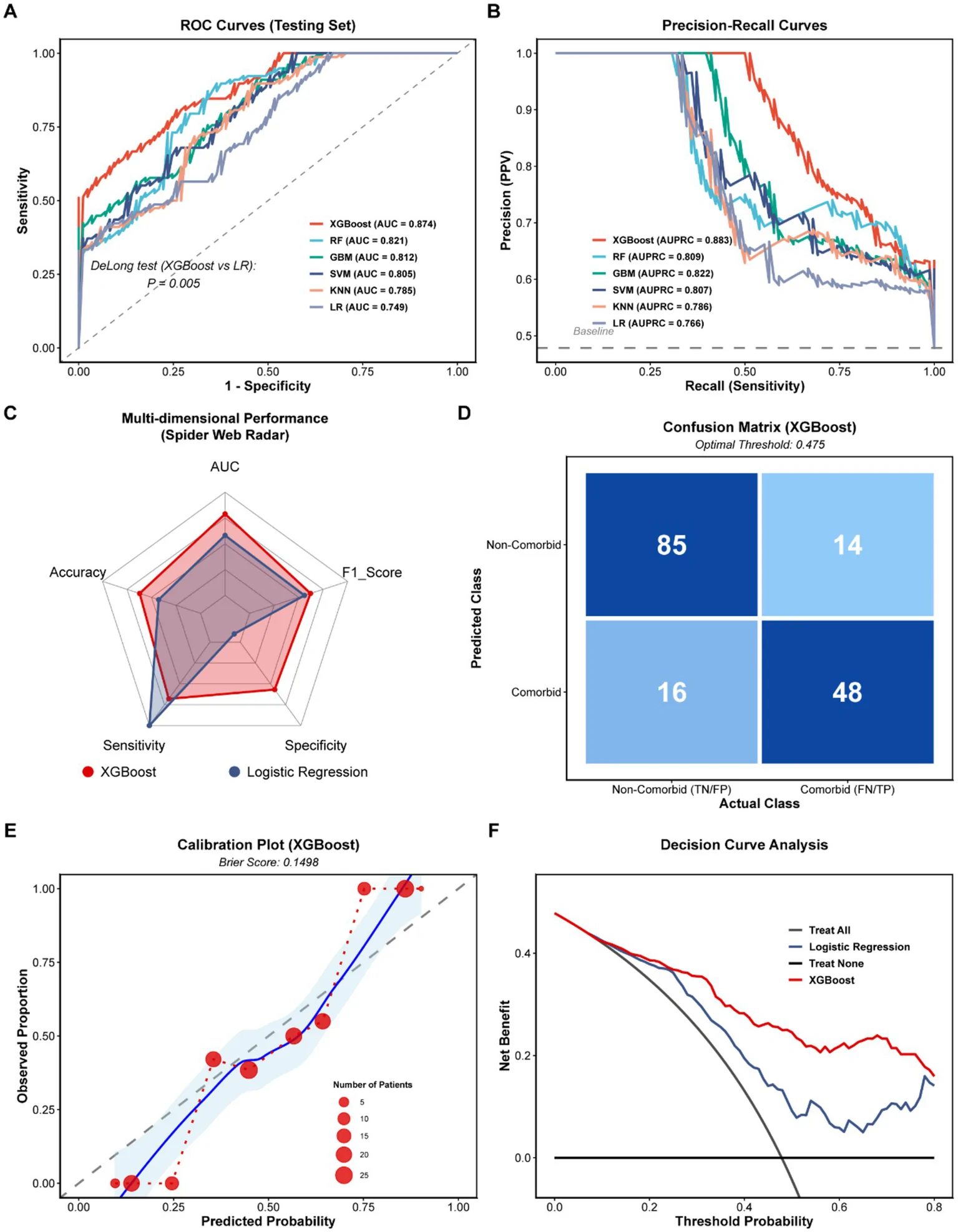
Performance evaluation of machine learning models in the testing set. **(A)** Receiver operating characteristic (ROC) curves of six machine learning models, with the DeLong test indicating potential differences in discriminatory ability. **(B)** Precision-recall (PR) curves evaluating model precision under potential class imbalance. **(C)** Spider web radar chart comparing multi-dimensional performance metrics between the XGBoost and logistic regression models. **(D)** Confusion matrix of the XGBoost model at the optimal threshold. **(E)** Calibration plot of the XGBoost model, with the blue Loess smoothing line suggesting the agreement between predicted probabilities and observed proportions. **(F)** Decision curve analysis (DCA) evaluating the potential clinical net benefit across different threshold probabilities.

Figure 2D presents the confusion matrix of the XGBoost model, which was obtained based on the optimal classification threshold of 0.475. The matrix shows that the number of true positive cases (*n* = 48 out of 64 actual comorbid cases) and true negative cases (*n* = 85 out of 99 actual non-comorbid cases) were relatively well balanced with relatively limited false negative and false positive predictions.

In addition to discrimination, model calibration was evaluated to verify the reliability of the predicted probabilities. Sample size weighted bubbles are plotted in the calibration plot of the XGBoost model (Figure 2E), along with a Loess smoothing line. As seen in the plot, predicted probabilities were closely fitted around the core probability range (between 0.25 and 0.75) with an acceptable Brier score of 0.1498. The deviations seen at the extreme tails of the probability distribution may be due to the small number of patients in the respective risk bins (small bubble size). Finally, Decision Curve Analysis (Figure 2F) shows that the use of the XGBoost model to inform clinical decision making may have a higher net clinical benefit than either the “treat-all” or “treat-none” groups as well as the traditional LR model specifically across the threshold probability range of 15 to 75%, suggesting that at these thresholds, targeted clinical actions (e.g., heightened PACU monitoring, optimized preventive analgesia) could be clinically justified.

### 3.3 Global SHAP analysis and non-linear feature dependence

After determining the predictive power of the XGBoost model, the SHAP framework was used to interpret the impacts of the global features on the prediction of the comorbidity. The SHAP summary plot (Figure 3A) illustrates how each variable affects the model’s prediction in a specific direction. The bar chart (Figure 3B) shows the mean absolute SHAP values, which are used to rank these features. Preoperative cortisol (mean SHAP 1.396) and preoperative melatonin (mean SHAP 1.279) were the top two drivers followed by the preoperative ISI score (0.430) and age (0.311). Based on this ranking, markers of neuroendocrine stress and sleep–wake regulation are likely to be central to the comorbidity network, acting preoperatively.

**Figure 3:**
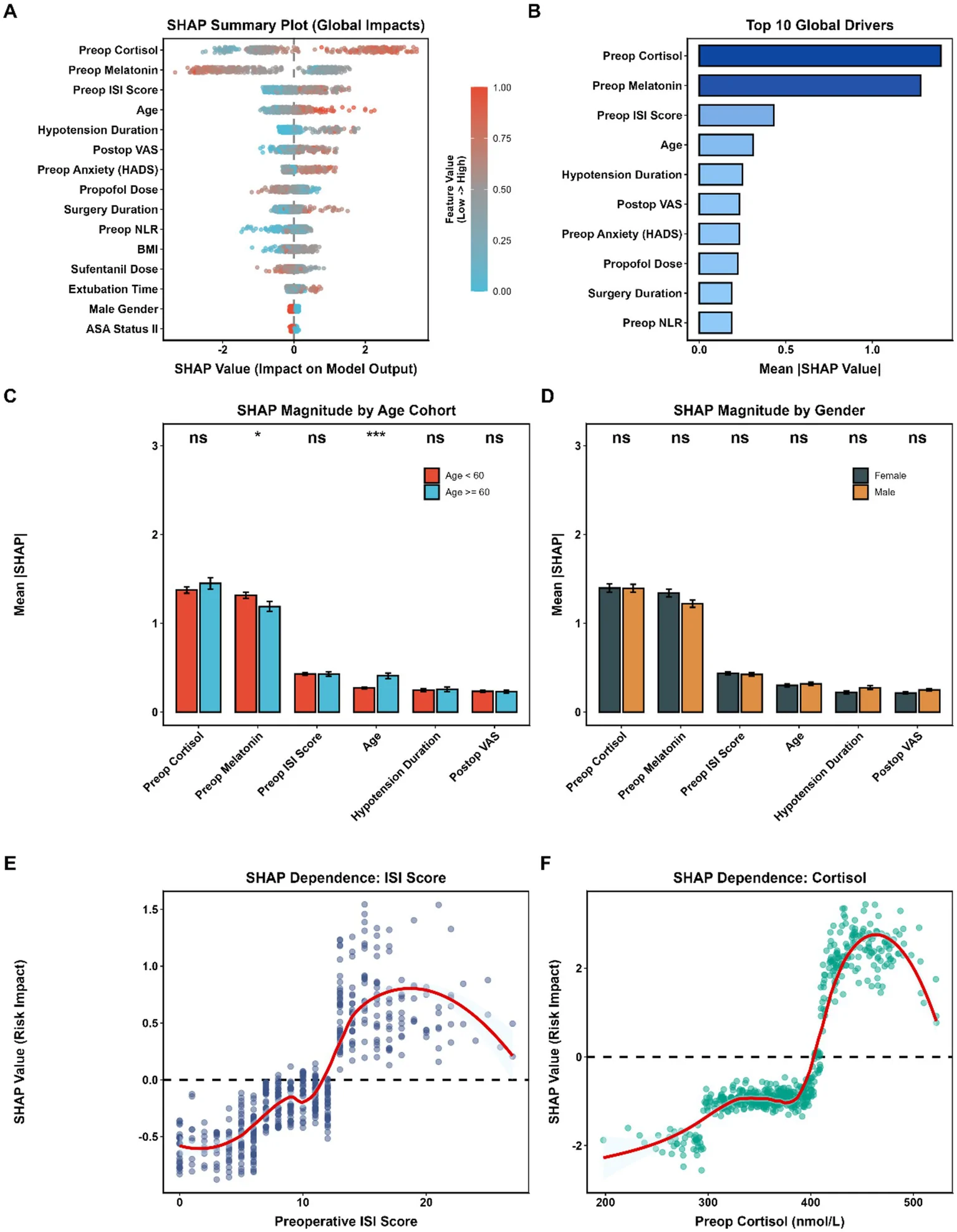
Global SHAP analysis indicating potential comorbidity drivers. **(A)** SHAP summary plot (beeswarm) showing the distribution of SHAP values for each feature across the cohort. **(B)** Bar chart ranking the top 10 global features based on mean absolute SHAP values. **(C,D)** Bar charts comparing mean absolute SHAP values stratified by age cohort **(C)** and gender **(D)**, with statistical significance indicated. **(E,F)** SHAP partial dependence plots for preoperative ISI score and preoperative cortisol, illustrating their marginal impacts and potential non-linear relationships with the model output.

SHAP magnitudes of the top features were stratified by age and gender cohort to investigate possible demographic heterogeneity. Although the majority of features had a relatively constant importance across age groups, the importance of the age variable itself was much higher in patients >60 years of age (*p* < 0.001) indicating a greater vulnerability in this age group (Figure 3C). However, gender-specific analyses (Figure 3D) showed no significant differences among the main features, suggesting that the drivers of this comorbidity may be sex-independent.

To understand the marginal effects of the important continuous variables, SHAP partial dependence plots were created. The effects of preoperative ISI and cortisol are not necessarily linear, as shown in Figures 3E,F; instead, a potential non-linear threshold effect is observed. For example, the SHAP value for the ISI score intersects the zero-risk line at approximately 12–13 points (Figure 3E), and the risk associated with preoperative cortisol levels rises sharply at around 400 nmol/L (Figure 3F). The slight downward curve observed at the high end of both fitted curves is noted, but this may be due to the small number of patient samples in these extreme ranges of data (edge artifacts from the smoothing algorithm) and may not necessarily be indicative of a biological protective effect. The observed non-linear thresholds also explain the differences in performance between traditional linear models and the ability to capture phenotypes of high risk, as described in the previous section.

### 3.4 Local SHAP analysis and individualized prediction trajectories

While the global SHAP analysis revealed the general mechanisms, the clinical expression of the comorbidity varies significantly among individuals. Local SHAP analysis was conducted to elucidate the personalized micro-trajectories of specific patients. Individual force plots were created to illustrate which top 10 features were contributing to the model’s prediction being pushed up (increasing risk, red) or down (decreasing risk, blue). As illustrated in Figure 4A, high preoperative cortisol (476 nmol/L, SHAP +2.53), low preoperative melatonin (2.2 pg./mL, SHAP +0.70) and severe preoperative insomnia (ISI = 26, SHAP +0.29) were the primary factors contributing to a positive prediction in a representative high-risk comorbid patient. By contrast, Figure 4B shows a “pure insomnia” patient (PID Only) with a high preoperative ISI score (17, SHAP +0.55), but who did not have emergence agitation because of the protective factors of low preoperative cortisol (338.1 nmol/L, SHAP −0.87), normal melatonin (15.3 pg./mL, SHAP −0.69), and well controlled postoperative pain (VAS = 2, SHAP −0.65). The contrast shows the possible compensation situation in which favorable clinical parameters may compensate for the risks of a single risk factor.

**Figure 4:**
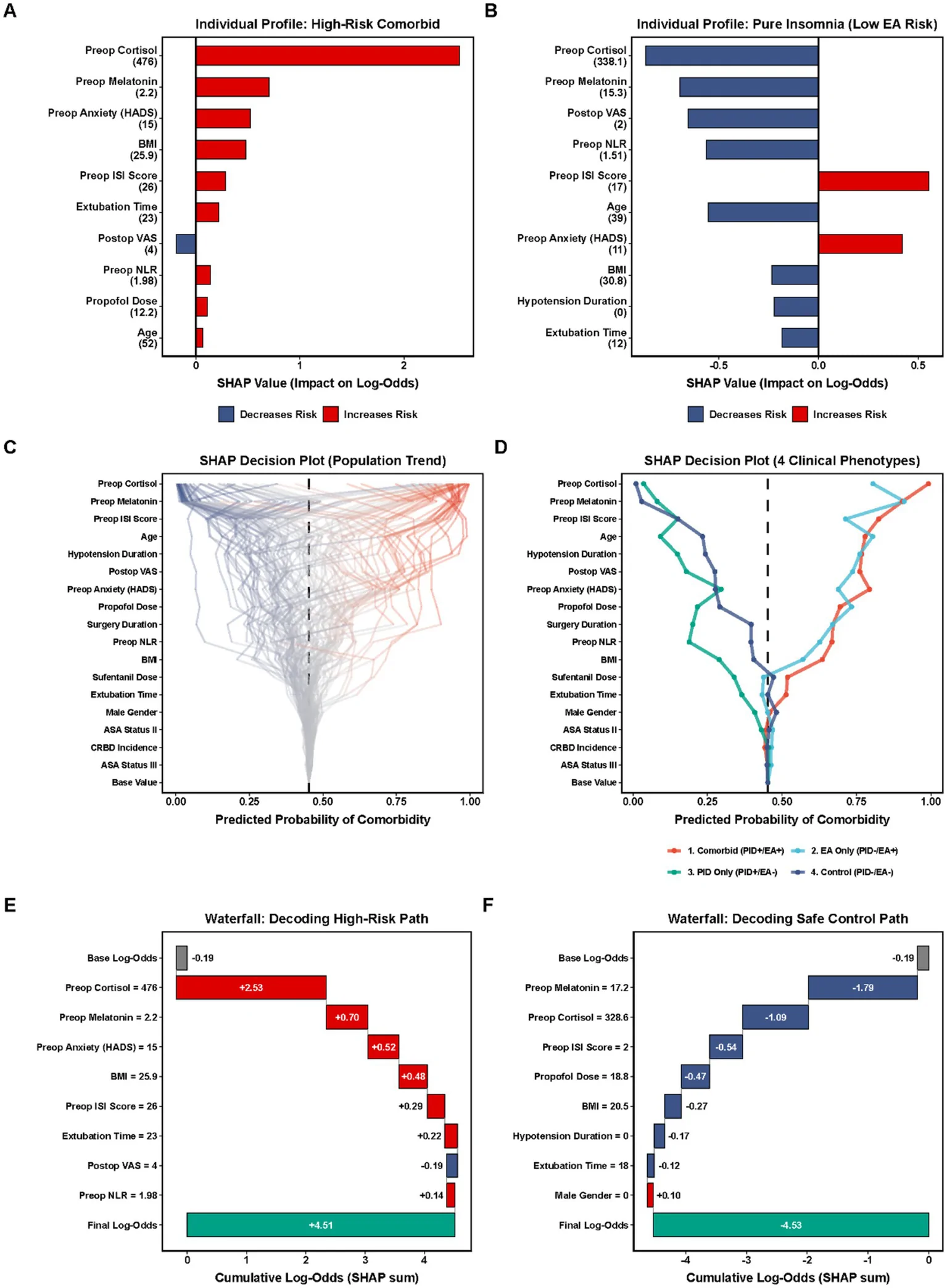
Local SHAP analysis and individualized prediction trajectories. **(A,B)** Individual force plots showing the feature contributions pushing the prediction up or down for a representative high-risk comorbid patient **(A)** and a pure insomnia patient **(B)**. **(C)** SHAP decision plot presenting the cumulative prediction trends for a random population sample. **(D)** Decision plot comparing the predictive trajectories of four typical clinical phenotypes. **(E,F)** Waterfall plots detailing the step-by-step accumulation of log-odds for a high-risk comorbid path **(E)** and a safe control path **(F)**.

SHAP decision plots were also created to further illustrate the dynamic risk accumulation process. Figure 4C shows the trend of the population, with the various ways in which 100 patients sampled at random depart from the base probability. In Figure 4D only the trajectories of four typical clinical phenotypes have been isolated. The plot shows that the four patients start with a shared base value, but diverge sharply upon encountering critical variables (e.g., cortisol and melatonin), ultimately resulting in distinct risk probabilities.

Waterfall plots were used to quantify the exact contribution of these variables step-by-step. In Figure 4E, the cumulative log-odds continually grew from the baseline to a high final probability, largely due to a successive increase in risk factors. In contrast, Figure 4F shows the safe control path with increasingly negative (safe) cumulative log-odds over the course of sequential protective factors (e.g., adequate melatonin and low cortisol). This individualized analysis indicates that the PID-EA comorbidity is not caused by one determinant alone but by a net effect of multiple determinants, both preoperative and perioperative that interact.

### 3.5 Identification of non-linear clinical thresholds via SHAP dependence

SHAP dependence plots were also created to gain insights into the ongoing effects of the different variables and to find potential clinical thresholds. These dependence plots estimate the change in the predicted risk overall, for the entire cohort, as the value of a particular feature changes, while the other features are kept constant, whereas the previous local analysis showed the various combinations of risks in an individual. When interpreting model SHAP values, it is crucial to remember that SHAP values are statistical associations and variable contributions within the model and not directly causal.

The marginal effect of the preoperative ISI score is illustrated in Figure 5A, demonstrating a strong positive correlation (Spearman rho = 0.87, *p* < 0.001). The SHAP value may enter the positive risk space when the ISI score goes above ~12, in a non-linear manner as suggested by the smoothing of the Loess line. In a similar fashion, Figure 5B shows that a S-shaped association exists for preoperative cortisol (Spearman rho = 0.84, *p* < 0.001). The risk impact becomes significantly greater and the point of zero risk is at about 400 nmol/L, which may be a critical level of neuroendocrine stress. On the other hand, the preop melatonin (Figure 5C) showed a negative correlation (Spearman rho = −0.68, *p* < 0.001). As seen on the plot, if melatonin level is above ~15 pg./mL, it tends to be protective (SHAP values < 0), but if below, it tends to be harmful.

**Figure 5:**
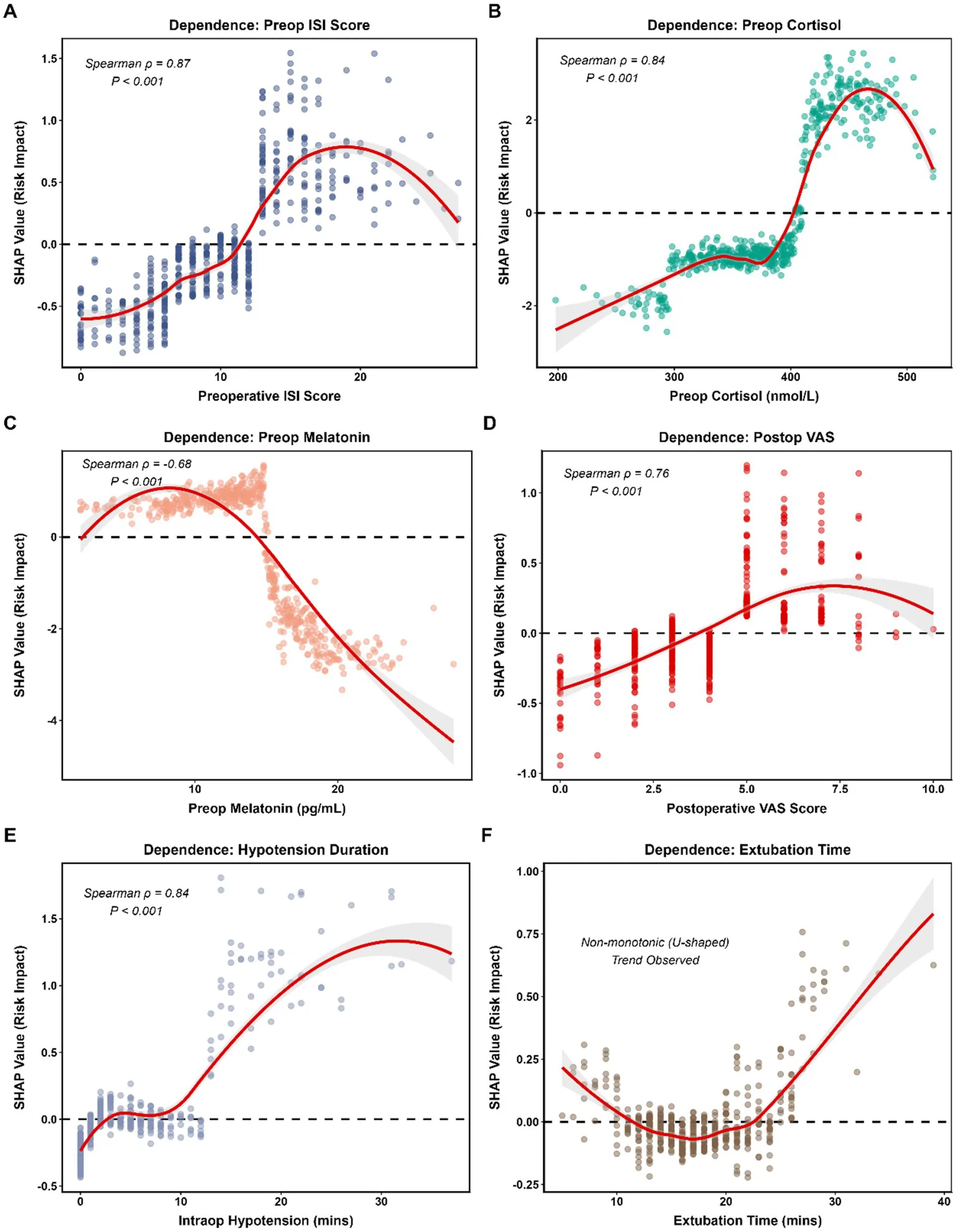
SHAP dependence plots illustrating the marginal effects of individual clinical features. **(A–F)** Dependence plots for Preoperative ISI Score, Preoperative Cortisol, Preoperative Melatonin, Postoperative VAS Score, Hypotension Duration, and Extubation Time. The red Loess smoothing lines illustrate the dynamic changes in SHAP values as feature values increase, which may suggest potential clinical risk thresholds or non-monotonic trends.

Non-linear risk accumulation was also shown for perioperative stressors. The dependence plot for postoperative VAS scores (Figure 5D) indicates a risk transition point at about 4–5 (Spearman rho = 0.76, *p* < 0.001). In the case of intraoperative hypotension duration, the model suggests that 10–12 min or more of hypotension is associated with increasing risk of the comorbidity (Figure 5E). Interestingly, the dependence plot for extubation time (Figure 5F) reveals a non-monotonic, U-shaped relationship rather than a linear trend. The lowest risk impact appeared to cluster within an intermediate time window (around 15 to 25 min), while both premature and prolonged extubation times were associated with positive SHAP values.

Finally, it should be noted that the slight downward or upward tailing seen at the extreme ends of the Loess smoothing curves in several plots (e.g., the right tails of the curves in Figures 5A,B,D) is probably due to edge artifacts. Usually these changes are due to smoothing algorithm effects on the few data points at the extremes of the clinical variables and do not reflect a real shift in clinical risk.

### 3.6 Decoding comorbidity mechanisms via SHAP interaction networks

SHAP interaction analyses were also performed to explore the possible synergies between the clinical variables. The independent marginal effects are described in the previous section, but it is important to appreciate how factors interact to assess the complicated comorbid phenotype. The global interaction matrix in Figure 6A shows the estimated strengths of interaction specifically among the top 7 globally ranked features to prevent visual overcrowding and computational noise from lower-tier variables. The most outstanding interaction signals were obtained from the combination of the ISI score before surgery with the VAS after surgery, and of the ISI score before surgery with the cortisol level before surgery.

**Figure 6:**
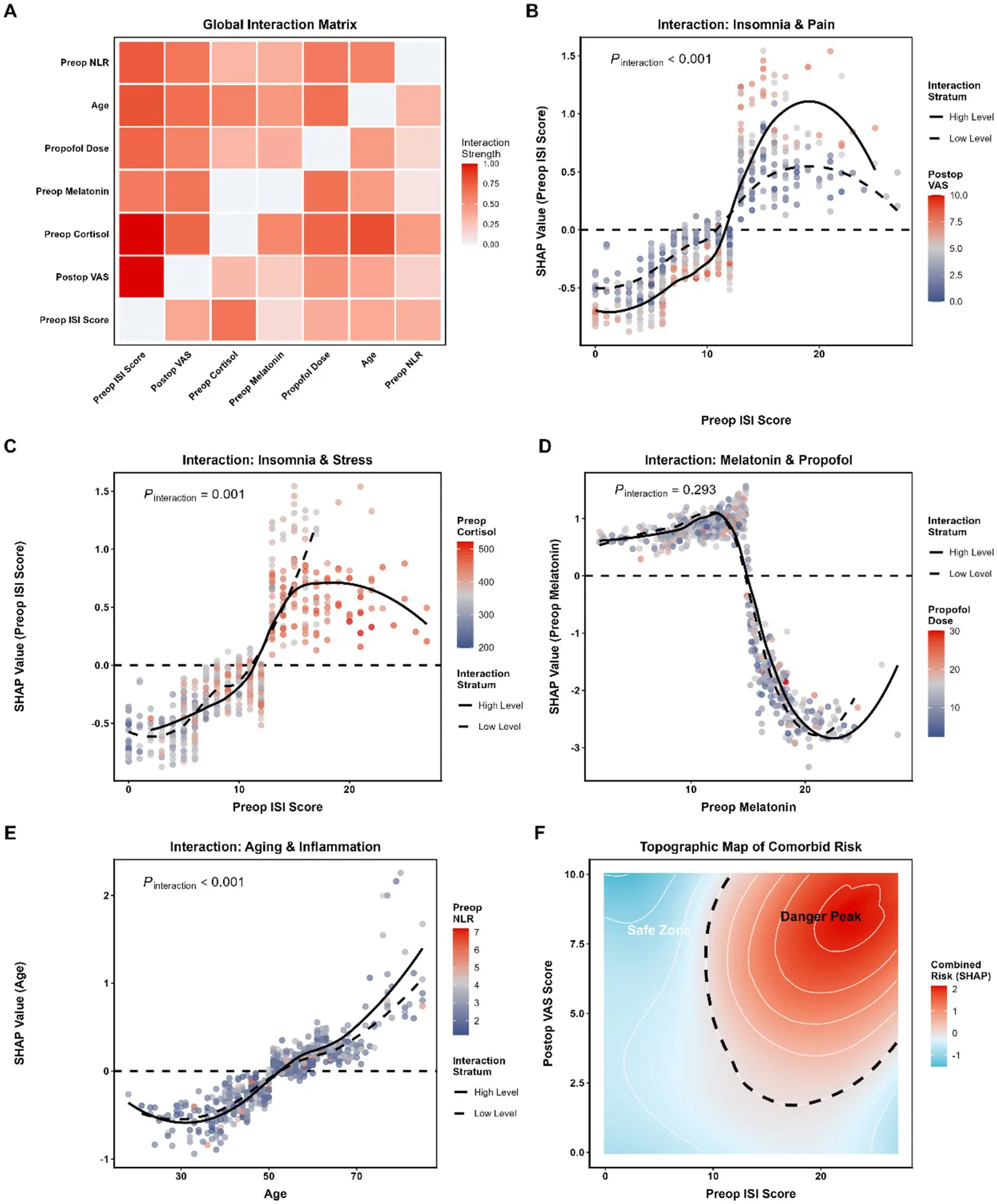
SHAP interaction networks suggesting potential synergistic effects among features. **(A)** Global interaction matrix showing the estimated interaction strength between pairs of top features. **(B–E)** Interaction scatter plots for selected feature pairs. The solid and dashed lines represent Loess smoothing curves stratified by the interacting variable (high vs. low levels), which may indicate interaction effects on the SHAP values. **(F)** A 2D topographic map illustrating the combined risk (total SHAP value) distribution jointly driven by preoperative ISI and postoperative VAS scores.

In order to visualize these particular synergies, interaction scatter plots were created using Loess smoothing lines stratified into high and low levels of the interaction variable. A significant statistical interaction was found between the pre-op ISI and post-op VAS (p interaction < 0.001) as shown in Figure 6B. The risk impact of insomnia was more strongly related to higher postoperative pain (solid line) than to lower pain scores (dashed line). This indicates that lack of adequate pain control may exacerbate neurobehavioural risks found in a preexisting sleep disturbance. A similar synergistic relationship was observed between preoperative ISI and preoperative cortisol (Figure 6C, interaction *p* = 0.001), suggesting that there may be a multiplicative effect of the combination of neuroendocrine stress and sleep deprivation.

Interestingly, not all high-ranking variables demonstrated an interactive relationship. The relationship between the dose of propofol during surgery and the melatonin level before surgery is shown in Figure 6D. No significant interaction was found (P interaction = 0.293) and the stratified smoothing curves were more or less parallel. This suggests the possible protective effect of melatonin may not be dependent on intra-operative propofol dose. Note that the slight and minor increase in the smoothing curve in Figure 6D at the extreme right tail is probably an algorithmic edge artifact and not a physiological change from protective to detrimental effect.

In addition, Figure 6E demonstrates an age related vulnerability in combination with a systemic inflammatory response. The separation between the smoothed curves suggests that the risk of the comorbidity in particular in older age groups might be increased by the elevated preoperative NLR (P interaction < 0.001).

Lastly, a 2D topographic contour map was generated (Figure 6F) to present the overall clinical risk from the primary interaction. This is a map showing the compound SHAP risk based on the preoperative ISI and postoperative VAS scores. The dashed contour line is intended to illustrate how levels of insomnia prior to surgery and levels of pain post-surgery form a continuum that could quickly move a patient from a relatively safe state to a high-risk comorbid state.

## 4 Discussion

Preoperative insomnia disorder (PID) and emergence agitation (EA) are challenging perioperative complications that frequently occur together, and a better understanding of the common pathophysiological pathways is warranted (15). In the present study, the primary findings indicate that the XGBoost model achieved better internal discrimination than the specified main-effects logistic regression benchmark for identifying this clinical co-occurrence. Furthermore, variables such as preoperative cortisol and melatonin contributed substantially to the model’s predictions, while SHAP analysis revealed exploratory non-linear and non-additive prediction patterns among perioperative features.

Most of the previous models for predicting postoperative behavioral disturbances have been logistic regression models. However, these conventional methods inherently assume linear independence among clinical variables (16, 17). As is demonstrated in other areas of application of machine learning in anesthesia, our study indicates that the specific XGBoost algorithm utilized in this dataset may offer advantages in managing complex clinical associations compared to the tested logistic regression model. Consequently, XGBoost demonstrated acceptable discrimination and calibration and was able to better capture these non-linear patterns.

As for feature contribution, we found that cortisol and melatonin were key global drivers in our model, prior to the surgery. This is consistent with current studies that have proposed that dysfunction of the hypothalamic–pituitary–adrenal axis and disruption of circadian rhythms may increase a patient’s risk of postoperative neurocognitive dysfunction (18, 19). Furthermore, the SHAP dependence plots were non-linear, with higher risk impacts observed when pre-op cortisol levels exceeded approximately 400 nmol/L. It is important to stress that SHAP values are not biologically causal but instead are statistical associations and contributions of variables to the algorithmic model (20). Moreover, minor variations occurring at the two ends of the smoothing curves are probably due to algorithmic edge effects due to the lack of data points and are not true physiological turnovers.

A notable aspect of this research is the study of synergistic interaction mechanisms. Inadequate analgesia is generally considered to increase postoperative agitation (21). This concept was mathematically visualized in our interaction analysis, suggesting that severe postoperative pain may synergistically amplify the risk associated with preoperative insomnia. Clinically, this implies that patients identified with preoperative sleep disturbances should be prioritized for rigorous multi-modal analgesia protocols in the PACU to blunt this synergistic risk spike. Furthermore, the non-monotonic association of extubation time indicates potential hazards in both premature and delayed emergence. Translated into practice, adhering to an optimal extubation window (e.g., 15 to 25 min) may serve as a concrete operational target to minimize neurobehavioral turbulence, highlighting the tangible benefit of individualized anesthesia management.

This study has several limitations. First, the retrospective and single-center design restricts the generalizability of the findings, and the results may be influenced by inherent selection bias. Second, subjective measurement of preoperative insomnia was used with the Insomnia Severity Index questionnaire; measurement bias may have occurred as polysomnography was not used. Further prospective, multi-center studies with objective, perioperative monitoring will be required to examine the validity of these possible clinical thresholds and interaction networks. Third, there is inherent uncertainty regarding the temporal order and potential reverse causality of postoperative variables, particularly postoperative pain. Pain may contribute to EA, occur concurrently with EA, or be exceedingly difficult to assess reliably in patients who are already agitated in the PACU. Thus, postoperative VAS should be interpreted strictly as an associative feature rather than a preceding causal factor.

## 5 Conclusion

In this study, a machine learning framework integrating XGBoost and SHAP analysis was utilized to evaluate the predictive associations and model-based interaction patterns between preoperative insomnia disorder (PID) and emergence agitation (EA). The results indicate that the specified XGBoost model discriminated better than the benchmark logistic regression model for this complex comorbidity phenotype. SHAP dependence and interaction analyses revealed exploratory predictive cutoffs (such as an Insomnia Severity Index > 13 and high preoperative cortisol), a and indicated that other variables such as postoperative pain and inflammatory markers might exhibit interactive associations the risk of EA in patients with preexisting sleep problems. While this study has limitations due to its observational nature involving a single center, and therefore only shows statistical associations, it highlights specific clinical nodes where targeted, personalized interventions for perioperative risk management could be developed. Furthermore, the predictive importance of the Insomnia Severity Index should be interpreted with caution, given its dual role as both a diagnostic component for PID and a predictive feature. These possible associative patterns should be validated in further multi-center prospective studies.

## Data Availability

The raw data supporting the conclusions of this article will be made available by the authors, without undue reservation.
